# Entre algoritmos y vulnerabilidades sociales: cruces entre salud, computación y vida social

**DOI:** 10.1590/S0104-59702026000100041

**Published:** 2026-10-12

**Authors:** Paola Peniche Moreno

**Affiliations:** i Profesora y investigadora, Ciesas, Unidad Peninsular. Mérida – YUC – México. ppeniche@yahoo.com

**Figure:**
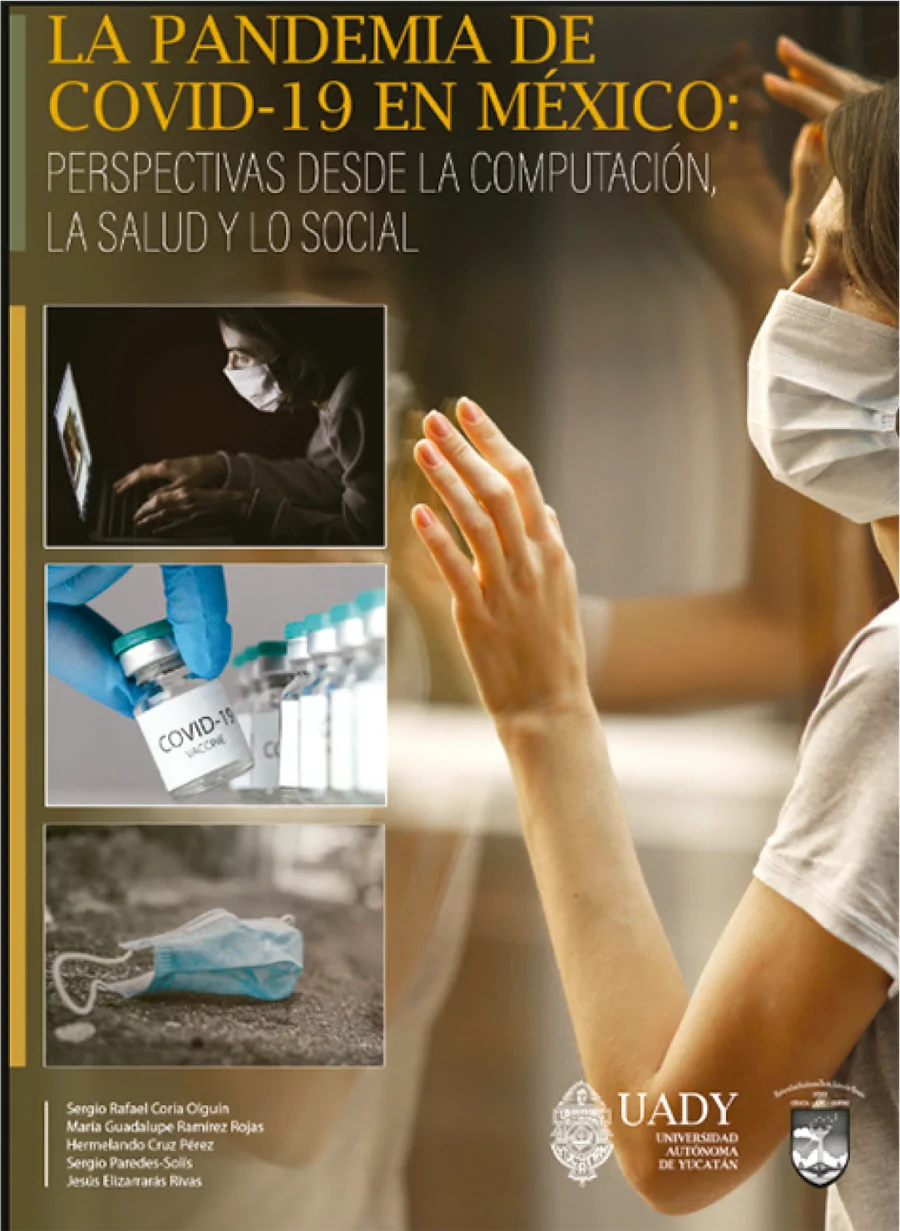

El libro *La pandemia de covid-19 en México: perspectivas desde la computación, la salud y lo social* (Olguín et al., 2025) propone una lectura multidisciplinaria del comportamiento del covid-19 en México, a partir del análisis de la base de datos publicada por la Secretaría de Salud. Con un enfoque cuantitativo, los autores exploran patrones clínicos, geográficos y sociodemográficos mediante técnicas de estadística descriptiva y aprendizaje automático, trabajando con registros de más de 18 millones de personas.

Debo confesar que, siendo científica social, enfrentarme a esa magnitud de datos y técnicas especializadas en computación y análisis estadístico resultó, a primera vista, abrumador. Sin embargo, los millones de registros analizados, lejos de ser solo números, son huellas de decisiones, vulnerabilidades y respuestas colectivas. Así, los resultados permiten visibilizar fenómenos sociales amplios, como la desigualdad y la marginación. Al entrecruzar computación, salud y ciencias sociales, los autores convierten los números en un espejo: reflejan cómo vivimos, enfermamos y, sobre todo, cómo nos enfrentamos colectivamente a la fragilidad de nuestro entorno social y sanitario.

Uno de los aspectos más relevantes de la obra reside en la articulación de enfoques provenientes de la ciencia de datos, la salud pública y las ciencias sociales, orientados al análisis de fenómenos sanitarios complejos. Desde esta convergencia disciplinaria, se propone una aproximación exploratoria que busca identificar regularidades significativas tanto a nivel individual como municipal.

La obra se estructura en ocho capítulos que desarrollan de forma progresiva un análisis multidimensional. Tras una introducción dedicada a los datos abiertos utilizados y a la motivación del estudio, el primer capítulo presenta el diseño metodológico, donde se explican las unidades de análisis, el alcance de la investigación y las técnicas empleadas. Los capítulos dos y tres ofrecen un panorama estadístico descriptivo, primero general y luego centrado en los casos positivos. A partir del capítulo cuatro, el análisis se vuelve más complejo con la incorporación de modelos de aprendizaje automático, el estudio de dinámicas cronológicas por municipio y la representación espacial a través de distintos tipos de mapas. El capítulo siete explora la relación entre la propagación del covid-19 y los perfiles sociodemográficos de los municipios, mientras que el capítulo final plantea una reflexión crítica sobre los desafíos del subregistro, la heterogeneidad territorial y las perspectivas futuras de investigación.

Quisiera centrar mi reflexión en cuatro elementos que atraviesan todo el libro: (1) el Estado como generador de información estadística y el papel de la ciencia en la política pública, (2) la relevancia de identificar patrones para enfocar el análisis de la vida colectiva, (3) la riqueza del nivel municipal para la política pública y el análisis social, (4) el valioso aporte que significa la clara identificación de las llamadas olas epidémicas.

Desde sus inicios, los Estados nacionales han tenido como preocupación central a la población y han recurrido a la información estadística como herramienta fundamental para su administración. En México, los primeros conteos poblacionales a finales del siglo XIX coincidieron con el fortalecimiento del Estado moderno y con el papel creciente de la ciencia en la formulación de políticas públicas (Peniche, 2024). Durante gran parte de los siglos XIX y XX, la producción científica estuvo estrechamente vinculada al Estado, orientada por agendas oficiales y alejada del escrutinio público. Esta relación comenzó a transformarse, en 2002, con la Ley Federal de Transparencia, y especialmente con la Política de Datos Abiertos de 2014 y la reforma constitucional en materia de transparencia, que promovieron la apertura sistemática de bases de datos gubernamentales (Chípuli-Castillo, 2022). Sin embargo, fue durante la pandemia de covid-19 cuando esta apertura adquirió una relevancia sin precedentes. Por primera vez, se difundió en tiempo casi real una gran cantidad de información sanitaria, lo que permitió la participación de investigadores y analistas independientes. Esta nueva dinámica supuso un giro en la relación entre Estado y ciencia, al exigir del campo académico no solo capacidad técnica, sino también una responsabilidad crítica frente al uso y sentido de los datos públicos.

Este libro asume esa responsabilidad y representa un punto de partida. Los resultados que aquí se presentan deben traducirse en productos concretos: análisis aplicados, insumos para el diseño de políticas públicas y estrategias específicas de prevención.

El segundo punto que destaco se refiere a la importancia de identificar patrones para orientar el análisis de la vida colectiva. En este sentido, el libro ofrece hallazgos significativos. La búsqueda de patrones no triviales implica una mirada crítica que permite reconocer relaciones sociales subyacentes y dinámicas de vulnerabilidad acumulada. Un ejemplo revelador es la asociación entre la categoría censal de ser indígena y una mayor probabilidad de desarrollar neumonía. Este vínculo plantea preguntas fundamentales: ¿qué condiciones estructurales permiten que una categoría estadística se convierta en un marcador epidemiológico? ¿Cómo se transforma una pertenencia cultural en un indicador de riesgo ante enfermedades respiratorias? Otro caso es la correlación entre municipios con bajos niveles de escolaridad y tasas elevadas de mortalidad por covid-19. Este patrón sugiere que la educación forma parte de un entramado más amplio de desigualdades, que incide directamente en la capacidad colectiva para enfrentar riesgos sanitarios.

Estos patrones complejos ofrecen insumos clave para comprender cómo interactúan las dimensiones sociales, económicas y territoriales en la configuración de la vulnerabilidad en contextos de crisis.

El tercer punto que me propuse desarrollar se refiere al valor del nivel municipal en la formulación de políticas públicas y en el análisis social. En este plano, el libro permite reflexionar sobre una de las grandes paradojas de la salud pública: mientras la propagación de las epidemias desborda fronteras, las estrategias para registrarlas, prevenirlas o mitigarlas se diseñan a partir de divisiones políticas y territoriales bien definidas, como países, estados o municipios (Peniche, 2024).

Dentro de esta tensión, los municipios emergen como unidades privilegiadas para observar las dinámicas socioculturales que inciden en los fenómenos epidémicos. Su relevancia no solo radica en su función político-administrativa, sino en su profundidad histórica como espacios donde se entrelazan relaciones sociales, prácticas culturales y desigualdades estructurales. Desde el siglo XIX, han sido clave para implementar reformas higienistas, canalizar intereses colectivos y organizar respuestas comunitarias frente a problemas sanitarios.

Uno de los principales aportes del libro es haber centrado buena parte del análisis estadístico en esta escala territorial, permitiendo identificar cómo interactúan las variables sociales y espaciales en la propagación y contención del covid-19. En particular, el análisis cronológico por municipios permite seguir con claridad el curso de las olas epidémicas, asociando su evolución con cambios en las políticas públicas y en las formas sociales de respuesta.

Durante la primera ola, en 2020, los municipios urbanos con alta movilidad (como los del Valle de México y Monterrey) concentraron el mayor número de casos, en un contexto de confinamiento estricto. En la segunda ola, a inicios de 2021, coincidente con la temporada invernal, se registró un aumento sostenido de contagios y muertes en zonas densamente pobladas, acompañado de un debilitamiento de las medidas restrictivas. En la tercera y cuarta olas (agosto de 2021 y enero de 2022), el impacto se desplazó hacia municipios rurales y marginados, donde se evidenciaron limitaciones estructurales para contener el virus. Finalmente, la quinta ola mostró un incremento en los casos positivos, pero con menor letalidad, en parte por el avance de la vacunación y por la adaptación social a las medidas preventivas.

En suma, este libro es una muestra de cómo el cruce entre ciencias computacionales, estadística y ciencias sociales puede ofrecernos herramientas para entender no solo la dinámica de una pandemia, sino las estructuras sociales que la condicionan. Los datos aquí analizados nos permiten ver más allá de los números: nos interpelan sobre la desigualdad, la capacidad estatal y la respuesta social. Este trabajo sienta las bases para futuras investigaciones y políticas públicas que reconozcan la complejidad social y territorial de los fenómenos sanitarios.

El formato digital de *La pandemia de covid-19 en México: perspectivas desde la computación, la salud y lo social* se encuentra disponible para descarga gratuita en la página de la librería de la Universidad Autónoma de Yucatán.

## Data Availability

No están en repositorio.
